# Risk of invasive meningococcal disease in children and adults with HIV in England: a population-based cohort study

**DOI:** 10.1186/s12916-015-0538-6

**Published:** 2015-12-09

**Authors:** Ruth D. Simmons, Peter Kirwan, Kazim Beebeejaun, Andrew Riordan, Ray Borrow, Mary E. Ramsay, Valerie Delpech, Samuel Lattimore, Shamez Ladhani

**Affiliations:** Immunisation Department, Public Health England, 61 Colindale Avenue, London, NW9 5EQ UK; HIV and STI Department, Public Health England, London, UK; Royal Liverpool Children’s Hospital, Liverpool, UK; Vaccine Evaluation Unit, Public Health England, Manchester, UK

**Keywords:** HIV, Invasive meningococcal disease, Serogroup, Vaccination, Relative risk

## Abstract

**Background:**

Recent studies have identified HIV infection as a potential risk factor for invasive meningococcal disease (IMD), suggesting that HIV-infected individuals could benefit from meningococcal vaccination to reduce their risk of this rare, but severe and potentially fatal infection. In the United Kingdom, as in most industrialised countries, HIV is not considered a risk factor for IMD.

**Methods:**

IMD incidence and relative risk by age group and meningococcal capsular group in HIV-positive compared with HIV-uninfected individuals was estimated through data linkage of national datasets in England between 2011 and 2013.

**Results:**

IMD incidence among persons diagnosed with HIV was 6.6 per 100,000 compared to 1.5 per 100,000 among HIV-negative individuals, with a relative risk of 4.5 (95 % CI, 2.7–7.5). All but one case occurred in adults aged 16–64 years, who had a 22.7-fold (95 % CI, 12.4–41.6; *P* <0.001) increased risk compared with the HIV-negative adults. IMD risk by capsular group varied with age. HIV-positive children and adolescents had a higher risk of meningococcal group B disease, while adults were at increased risk of groups C, W and Y disease. Most HIV-positive individuals had been born in Africa, had acquired HIV through heterosexual contact, and were known to be HIV-positive and receiving antiretroviral treatment at IMD diagnosis. The most common clinical presentation was septicemia and, although intensive care admission was common, none died of IMD.

**Conclusions:**

HIV-positive children and adults are at significantly increased risk of IMD, providing an evidence base for policy makers to consider HIV as a risk factor for meningococcal vaccination.

## Background

*Neisseria meningitidis*, commonly known as the meningococcus, is a major global cause of meningitis and septicaemia, and is associated with significant morbidity and mortality across all age groups [1]. Twelve distinct polysaccharide capsules have been described, including five that are responsible for almost all cases of invasive meningococcal disease (IMD) globally: A, B, C, W and Y [1].

In the United Kingdom, as in most of Europe, meningococcal groups B (MenB) and C (MenC) were previously responsible for nearly all IMD cases [2]. Invasive MenC disease, however, is now uncommon since routine vaccination against MenC was introduced in 1999, with most cases now occurring in unvaccinated adults who acquire the infection abroad [2]. MenB, therefore, has been responsible for 80–90 % of all IMD cases, with the highest incidence in infants (<1 year-olds) and toddlers (1–4 year-olds), and with a small secondary peak in adolescents and young adults [3]. Meningococcal groups W (MenW) and Y (MenY) are uncommon and mainly cause disease in older adults, although the UK is currently experiencing a national outbreak of MenW disease across all age groups, following endemic expansion of a single hypervirulent strain belonging to the ST-11 clonal complex [4].

Unlike older adults (≥65 year-olds), the vast majority of children and young adults who develop IMD are previously healthy. The only significant risk factors for IMD are complement deficiency and asplenia/splenic dysfunction [5]. These risk groups are currently advised to receive the meningococcal quadrivalent conjugate vaccine (MenACWY) and the recently licensed multi-component protein-based MenB vaccine (Bexsero®, Novartis, Basel, Switzerland). They may also be on penicillin prophylaxis, which should provide additional protection against IMD [5]. Both these vaccines will be included in the UK national infant and adolescent immunisation programmes, respectively, later this year [5].

Unlike other encapsulated bacteria, such as *Streptococcus pneumoniae* and *Haemophilus influenzae* type b (Hib), immunosuppression (primary or acquired) has not been considered a significant risk factor for IMD [5]. Although the British HIV Association (BHIVA) testing guidelines recommend the quadrivalent vaccine containing A, C, W and Y, for travellers considered to be at risk or in contact with these capsular groups [6], HIV-positive individuals are not recommended to receive any additional vaccinations against IMD apart from the scheduled routine MenC doses, irrespective of their degree of immunosuppression or HIV treatment status [7–13].

The objective of this study was to assess the risk of IMD by age and capsular group in persons diagnosed with HIV in England. Analysis was performed using national data for the three most recent years, with the aim of developing an evidence base for recommending meningococcal vaccination for this vulnerable group.

## Methods

### Meningococcal surveillance

Public Health England (PHE) conducts enhanced national surveillance of IMD and captures more than 95 % of laboratory-confirmed cases in England [3]. As part of the enhanced surveillance, the PHE Meningococcal Reference Unit (MRU) provides a national service for species confirmation and grouping/typing of invasive *N. meningitidis* isolates, which are not routinely performed by National Health Service (NHS) hospital laboratories. The MRU also offers free PCR testing for meningococcal DNA in clinical specimens (e.g. blood, cerebrospinal fluid, joint fluid, pleural fluid), which are routinely submitted by NHS hospital laboratories throughout England [3]. Species confirmation and capsular group determination were performed as described previously [14]. Since 1 January 2011, all laboratory-confirmed cases are followed-up by requesting the patient’s general practitioner (GP) to complete a short questionnaire requesting information on risk factors, co-morbidities, clinical presentation, complications and outcome of IMD.

### HIV surveillance

In addition to the information collected through GP questionnaires, all confirmed cases of IMD were matched to all persons newly diagnosed with HIV between 1981 and 2013, collected through a comprehensive national cohort of persons presenting for an HIV test across all testing facilities in England and subsequently accessing HIV care. Details of the HIV surveillance systems are detailed on the PHE website [15–17]. Cases of meningococcal disease were linked to persons diagnosed with HIV using soundex code (pseudo-anonymised coding of surname), initial, date of birth, sex and region of diagnosis. Data available on persons diagnosed with HIV are estimated to represent 76 % of all persons living with HIV, with around a quarter of persons living with HIV currently unaware of their infection. Reports of new HIV diagnoses for the study period, January 2011 to December 2013, are likely to be complete, with minimal reporting lag after two years.

Ethics agreement for this work is not required under the provisions in regulation 3 of *The Health Service (Control of Patient Information) Regulations 2002*, which authorises patient information to be processed by persons employed or engaged for the purposes of the health service or other persons employed or engaged by a government department or other public authority in communicable disease surveillance or associated with other risks to public health [18]. All data collected was under the remit of communicable disease surveillance.

### Population

The study population consisted of all persons with confirmed IMD in England between January 2011 and December 2013. IMD cases were matched to persons diagnosed with HIV to identify all potential co-infected persons using soundex, initials, date of birth and sex. Confirmed meningococcal cases who were not identified by their GPs to be HIV-positive and who did not link to the HIV database were considered to be HIV-uninfected.

### Definitions

Invasive meningococcal disease was defined as either isolation of *N. meningitidis* or DNA detection by PCR from a normally sterile body site [3].

A CD4 cell count reported within 90 days either side of HIV diagnosis was defined as the CD4 count at HIV diagnosis. A CD4 cell count below 350 cells/mm^3^ was defined as the time after which HIV treatment should have been commenced [6]. Therefore, any person with a CD4 cell count below 350 cells/mm^3^ was defined as having been diagnosed late. The CD4 cell count and ART status at IMD diagnosis among those co-infected was included if within 90 days of IMD diagnosis.

### Statistical analysis

Statistical analyses were performed using STATA version 10 (Stata Corp., College Station, TX, USA). IMD incidence for persons diagnosed with HIV was calculated using the national cohort of persons diagnosed and living with HIV [16]. IMD incidence for the general population was calculated using population denominators from the Office for National Statistics (www.statistics.gov.uk). The overall and age-specific HIV-uninfected population were defined as the difference between the general population and the number of diagnosed HIV-positive persons. Incidence was calculated for all IMD, capsular group-specific (B, C, W and Y) and vaccine-preventable (B and ACWY) IMD.

Relative risk of IMD in HIV-positive compared with HIV-uninfected individuals was calculated using the incidence of these measures in co-infected and uninfected persons over the study period. Rates were compared using Pearson’s *x*^2^ test. Proportions were compared using the *x*^2^ test or Fisher’s exact test, as appropriate. Using a backwards logistic regression model, we examined factors associated with being co-infected with IMD and HIV. Variables included sex, age group, capsular group and clinical presentation. All confidence intervals (CI) are at the 95 % significant level.

## Results

During 2011–2013, there were 2,353 confirmed IMD cases in England. The highest number of cases occurred in infants (<1 year-olds) who accounted for 21 % (499/2,345) of cases over the three-year period. IMD cases declined after the first year of life, although children less than 5 years old still accounted for nearly half of all cases (1,145/2,345, 49 %). A smaller peak was observed in adolescence, starting at 16 years and peaking at 19 years, before declining. MenB was responsible for 92 % (1,266/1,371) of cases in <16 year-olds, 68 % (480/703) in 16–64 year-olds and 41 % (96/237) in ≥65 year-olds. Meningococcal groups A, C, W and Y were responsible for 7.7 % (n = 105), 32 % (n = 222) and 59 % (n = 141) cases in these age groups, respectively.

### Demographics

Co-infection with IMD and HIV was identified in 14 cases. Eleven were known to be HIV-positive prior to IMD with a median time of 6.5 (IQR, 2.5–10.6) years between HIV diagnosis and IMD diagnosis, two were diagnosed with HIV when they developed IMD, and one developed IMD one year prior to being confirmed as HIV-positive. All but one IMD case (93 %) among HIV-positive individuals were in adults aged 16–64 years (Table 1).

**Table 1:** Distribution of persons with laboratory-confirmed invasive meningococcal disease (IMD) by HIV status in England during 2011–2013

	Persons diagnosed with HIV		Uninfected persons	
	n = 14	%	n = 2,339	%
Age at meningococcal infection (years)				
<16	1	7.1	1,395	59.8
16–24	3	21.4	314	13.5
25–44	5	35.7	159	6.8
45–65	5	35.7	225	9.7
≥65	0	0	238	10.2
Not reported			8	
Median age at IMD (IQR)	32	(24–48)	5	(1–29)
Sex				
Male	7	50	1,166	50.2
Female	7	50	1,159	49.8
Not reported			14	
Year of meningococcal diagnosis				
2011	6	42.9	880	37.6
2012	3	21.9	738	31.6
2013	5	35.7	721	30.8
Region of meningococcal diagnosis				
East of England	0	0	178	7.6
East Midlands	1	7.1	205	8.8
London	7	50.0	329	14.1
North East	0	0	156	6.7
North West	4	28.6	459	19.6
South East	2	14.3	307	13.1
South West	0	0	226	9.7
West Midlands	0	0	237	10.1
Yorkshire and the Humber	0	0	242	10.3
Meningococcal group				
A	0	0	1	0.0
B	4	28.6	1,841	78.7
C	3	21.4	81	3.5
W	2	14.3	146	6.2
Y	5	35.7	235	10.0
Other/non-groupable	0	0	35	1.5
Died				
Yes	0	0	150	6.4
No	14	100	2,189	93.6

### Incidence

The mean annual IMD incidence during 2011–2013 was 6.6 per 100,000 persons diagnosed with HIV compared with 1.5 per 100,000 among HIV-uninfected persons (relative risk, 4.5; 95 % CI, 2.7–7.5; *P* <0.001) (Table 2). The higher risk was observed for all three calendar years and across all age groups except older adults, where no IMD cases occurred in the HIV-positive cohort. When analysed by capsular group, the relative risk of IMD in adults was significantly higher for MenC, MenW and MenY (Table 3). An increased risk for MenB disease was only observed among adolescents and young adults (16–24 year-olds). For the other capsular groups, MenACWY were responsible for 31 % (212/690) of IMD cases in HIV-uninfected adults (16–64 year-olds) compared with 77 % (10/13) in those diagnosed with HIV, who had a 22.7-fold (95 % CI, 12.4–41.6; *P* <0.001) increased risk of IMD caused by these capsular groups.

**Table 2:** The incidence of invasive meningococcal disease (IMD) and relative risk by demographics for persons diagnosed with HIV and for the uninfected population in England during 2011–2013

	Persons diagnosed with HIV			Uninfected population					
	Cases	Person-years	Incidence	Cases	Person-years	Incidence	Relative risk	95 % CI	*P* value
Overall	14	213,725	6.6	2,339	160,252,992	1.5	4.5	(2.7, 7.5)	<0.001
Age at meningococcal infection (years)									
<16	1	2,415	41.4	1,395	30,366,823	4.6	9.0	(1.3, 64.0)	0.008
16–24	3	6,703	44.8	314	18,760,191	1.7	26.5	(8.6, 81.8)	<0.001
25–44	5	113,183	4.4	159	43,536,541	0.4	11.8	(5.0, 27.9)	<0.001
45–65	5	84,462	5.9	225	40,505,320	0.6	10.4	(4.4, 24.9)	<0.001
≥65	0	6,962	0.0	238	27,084,317	0.9			
Not reported				8					
Sex									
Male	7	142,479	4.9	1,166	78,858,090	1.5	3.3	(1.6, 6.9)	<0.001
Female	7	71,246	9.8	1,159	81,394,902	1.4	6.9	(3.3, 14.4)	<0.001
Not reported				14					
Year of meningococcal diagnosis									
2011	6	67,649	8.9	880	53,039,551	1.7	5.3	(2.4, 11.8)	<0.001
2012	3	71,316	4.2	738	53,422,384	1.4	3.0	(1.0, 9.4)	0.043
2013	5	74,760	6.7	721	53,791,057	1.3	5.0	(2.1, 11.9)	<0.001

**Table 3:** Incidence of invasive meningococcal disease (IMD) in adults and relative risk by HIV status and capsular group in England during 2011–2013

		Incidence of meningococcal				
Capsular group	Age group (years)	Persons co-infected with HIV	Uninfected persons	Rate ratio	95 % CI	*P* value
All	All	6.6	1.4	4.5	(2.7, 7.7)	<0.001
	16–24	44.8	1.7	26.8	(8.7, 82.8)	<0.001
	25–44	4.4	0.4	11.9	(5.0, 28.2)	<0.001
	45–64	5.9	0.6	10.5	(4.4, 25.1)	<0.001
ACYW	All	4.7	0.3	16.0	(8.7, 29.6)	<0.001
	16–24	29.9	0.4	73.7	(18.8, 289.4)	<0.001
	25–44	3.5	0.1	26.6	(10.3, 68.5)	<0.001
	45–64	4.7	0.2	21.8	(8.4, 56.9)	<0.001
B	All	1.9	1.1	1.6	(0.6, 4.3)	0.32
	16–24	14.9	1.3	11.8	(1.7, 83.6)	0.002
	25–44	0.9	0.2	3.7	(0.5, 26.1)	0.16
	45–64	1.2	0.3	3.5	(0.5, 24.4)	0.19
C	All	1.4	0.05	27.1	(8.9, 82.4)	<0.001
	16–24	-	0.1	-	-	-
	25–44	1.8	0.05	35.1	(9.4, 131.4)	<0.001
	45–64	1.2	0.03	32.0	(4.8, 212.8)	<0.001
Y	All	2.3	0.1	15.8	(6.7, 37.7)	<0.001
	16–24	14.9	0.2	73.7	(10.7, 509.9)	<0.001
	25–44	0.9	0.1	15.4	(2.3, 105.3)	<0.001
	45–64	3.6	0.1	32.0	(10.7, 95.6)	<0.001
W	All	0.9	0.1	10.2	(2.6, 40.5)	<0.001
	16–24	14.9	0.1	107.7	(15.8, 736.3)	<0.001
	25–44	0.9	0.02	35.1	(5.4, 227.2)	<0.001
	45–64	0.0	0.1	-	-	-

In children, although there was only a single case of MenB in three years, this still constituted an increased relative risk of 10.0 (95 % CI, 1.4–71.1; *P* = 0.005) for invasive MenB disease compared with HIV-uninfected children.

### Risk factors in persons diagnosed with HIV

Of the 14 HIV-positive individuals who developed IMD, 71 % had acquired HIV through heterosexual contact, 14 % through mother-to-child transmission (MTCT) and 14 % were men who have sex with men (MSM) (Table 4). IMD incidence was highest in those infected through MTCT (44.8 per 100,000 MTCT), compared with 9.4 per 100,000 heterosexuals diagnosed with HIV and 2.1 per 100,000 MSM.

**Table 4:** Characteristics of individuals co-infected with HIV and invasive meningococcal disease (IMD) in England during 2011–2013

Age group at IMD (years)	Group	Time between HIV and IMD diagnosis	Time between ART and IMD	CD4 <350 cells/mm^3^ at HIV diagnosis	CD4 at IMD <350 cells/mm^3^	HIV risk	Region of birth	Year of arrival	Clinical presentation	ICU admission
<16	B	2 years	2 years	N	N	MTCT	Africa	2008	Septicaemia	N
16–24	W	13 years	-	N/A	N/A	MTCT	Africa	2000	Pneumonia	Y
16–24	B	After IMD	After IMD	Y	N/A	MSM	Asia	2008	Men & Sep	Y
16–24	Y	2 years	2 years	Y	N	HET	Africa	2004	Men & Sep	Y
25–44	Y	11 years	11 years	Y	Y	HET	Africa	2002	Septic arthritis	N
25–44	C	9 years	9 years	N	N	HET	Africa	2000	Septicaemia	N
25–44	W	8 years	8 years	N	N	HET	Africa	2004	Septicaemia	Y
25–44	B	5 years	4 years	Y	Y	HET	Africa	2004	Septicaemia	N
45–64	C	5 years	At time of IMD	N	N/A	MSM	UK	-	Septicaemia	N
45–64	Y	16 years	2 years	N	Y	HET	UK	-	Pneumonia	N
45–64	Y	10 years	At time of IMD	Y	N/A	HET	Africa	1988	Septicaemia	Y
45–64	B	3 years	3 years	Y	Y	HET	Africa	2002	Meningitis	N
45–64	Y	At the time of IMD	After IMD	N	N	HET	Africa	1975	Men & Sep	Y
45–64	C	At the time of IMD	Not on ART	Y	Y	HET	UK	-	Septicaemia	N

*HET* heterosexual contact, *ICU* intensive care unit, *Men & Sep* meningitis and septicaemia, *MSM* men who have sex with men, *MTCT* mother-to-child transmission, *N* no, *N/A* not available, *Y* yes

Eight of the ten persons who acquired HIV through heterosexual contact and all persons infected through MTCT were born in Africa, resulting in an incidence of IMD among those diagnosed with HIV born in Africa being almost four times higher (IRR, 3.76; 95 % CI, 0.97–21.24) than that among those born in the UK (15.0 per 100,000 vs. 4 per 100,000).

Of the 11 persons diagnosed with HIV before developing IMD, the CD4 count, where reported, was <350 cells/mm^3^ in 50 % at HIV diagnosis (5/10) and at IMD diagnosis (4/8), and nearly all were on ART when they developed IMD (8/10). Of the five persons with a CD4 count of <350 cells/mm^3^ at HIV diagnosis, 75 % (3/4) continued to have a CD4 count of <350 cells/mm^3^ at IMD diagnosis, although all were receiving ART. The two adults diagnosed with HIV when they developed IMD had CD4 counts <350 and >350 cells/mm^3^, respectively, while the only case diagnosed with HIV more than one year after developing IMD had a CD4 count <350 cells/mm^3^.

Only 2 of the 14 cases had received a meningococcal vaccine prior to developing IMD, one was eligible for the MenC conjugate vaccine as part of the national MenC catch-up campaign during 1999–2000 but developed MenW disease, and the other had received the ACWY vaccine abroad and subsequently developed MenW disease. The three UK-born individuals were too old to have been eligible for any meningococcal vaccination.

The most common clinical presentation was septicaemia, followed by meningitis and pneumonia. Adjusting for age, gender and capsular group in a multivariable logistic regression model, clinical presentation was not significantly different for HIV-positive compared to HIV-uninfected cases. Although 6/14 (43 %) required intensive care support, no HIV infected people died of IMD.

## Discussion

During a period when ART was routinely available in England, persons diagnosed with HIV had a 4.8-fold increased risk of IMD compared with individuals not known to be HIV infected. This increased risk was almost entirely in adults aged 16–64 years (23-fold increased risk) and was significant for Men C, W and Y, while MenB was only significant in those aged 16–24 years. There was only one MenB case in children with HIV and no cases in ≥65 year-olds. Most patients were born outside the UK, were known to be HIV-positive and receiving ART at the time of infection, and had not received any meningococcal vaccination prior to developing IMD.

These findings are consistent with the most recent study from New York City (NYC) where the relative risk for IMD among people living with HIV/AIDS (PLHIV) during the ART era was 10.0 especially among those with a CD4+ count of less than 200 cells/mm^3^ [13]. Notably, we observed a 15.9-fold increased risk for MenC, MenW and MenY, which are all potentially preventable through immunisation with the MenACWY quadrivalent conjugate vaccine [19]. The NYC study also found these capsular groups to be more prevalent among HIV-infected adults with IMD (87 %) compared to the IMD-only group (72 %), although the difference was not statistically significant. An earlier study from the United States also reported a significantly higher risk of IMD in HIV-positive adults [7] and these findings were corroborated in a large South African study, where the age-adjusted relative risk of developing IMD was 11.3, with a two-fold increased risk of dying from the infection [8]. Other studies from developing countries with high HIV prevalence, however, have not found any significant associations with IMD [9–12].

In HIV-uninfected individuals, half of all IMD cases in England are diagnosed in children younger than 5 years and more than 90 % of cases in this age group are caused by MenB [3]. Although only one IMD case was diagnosed among children with HIV, this single case represented a significantly higher risk for HIV-positive children when compared with HIV-uninfected children.

In adolescents, a small peak in IMD coincides with teenagers entering higher education settings (typically universities), where they are exposed to lifestyle factors and behaviors that increase their risk of IMD, such as smoking (or being exposed to smoke), kissing, sharing drinking glasses, eating utensils or water bottles, and living in close quarters (e.g. dormitories) [20]. Overall, MenB is still responsible for the majority (85–90 %) of IMD cases in adolescents. In HIV-positive persons, the increased risk in invasive MenB disease was only observed among 16–24 year-olds. In contrast, the risk of MenW and MenY disease was significantly higher across all adult age groups. These two capsular groups are uncommon causes of IMD in England and generally considered to be less virulent than MenB or MenC, causing disease mainly in older adults who often have underlying co-morbidities [4, 21]. A notable feature of these two capsular groups is the atypical clinical presentation, including respiratory tract infections such as pneumonia, epiglottitis, cellulitis and septic arthritis [22]. If IMD is not considered in the differential diagnosis, then public health actions to offer rapid chemoprophylaxis and the MenACWY conjugate vaccine to close contacts (who may also be HIV-positive) could be delayed and lead to secondary cases.

Although we observed significantly higher rates of capsular group-specific IMD in HIV-positive individuals, there were only 14 cases diagnosed over the three-year period, equivalent to 0.5 % of all IMD cases. In this high-risk group, however, we found that most individuals had acquired HIV through heterosexual contact and the incidence in MSM was similar to the background rate. A recent MenC disease outbreak in NYC was reported among MSM and subsequently identified in other countries, including Germany and France, resulting in an offer of vaccination against MenC to MSM in these countries [13, 23–28]. In England, there were no cases of MenC disease in children, adolescents or young adults (<25 year-olds) with HIV over the three-year period and none among MSM of any age, highlighting the success of the current national MenC conjugate vaccination programme in providing both direct and indirect protection against MenC disease [29].

Unlike the US, MenB is responsible for most IMD cases in children and adults in England. The significantly higher risk of MenC, MenW and MenY disease among persons diagnosed with HIV in England is, therefore, intriguing. Most cases were known to be HIV-positive at IMD diagnosis and half of them continued to have low CD4 counts at IMD diagnosis, making them generally susceptible to serious bacterial infections, including the less virulent meningococcal capsular groups such as MenW and MenY. In persons known to be HIV-positive, we found no association between IMD and CD4 counts at HIV diagnosis, time since HIV diagnosis, CD4 counts at IMD diagnosis or receiving ART at IMD diagnosis. Given that most cases had been born outside the UK, it is likely that they were at higher risk of exposure to infection when they themselves, their close family or community contacts travelled to their country of origin. It is also possible that these cases socialise in highly-defined closed networks – either because of their HIV infection or because of their ethnic background – where the less common meningococcal capsular groups may be circulating.

### Limitations

Until now, except for complement deficiency and splenic dysfunction, HIV or any other immune deficiency has not been considered a significant risk factor for IMD [5]. In the current study, too, only 0.5 % of IMD cases were known to be HIV-positive. However, the IMD surveillance questionnaires were completed by GPs, who are not always aware of the diagnosis of HIV. It is, therefore, possible that HIV prevalence among IMD patients may be underestimated as demonstrated by the additional ascertainment through linkage with the national HIV database. In children, however, there were limited identifiers in the HIV database for linkage compared to adults and, therefore, some children with HIV and IMD may have been missed. It is also not possible to estimate the number of undiagnosed HIV-positive cases in the IMD cohort because the latter are not routinely tested for HIV. Furthermore, it is estimated that a quarter of persons estimated to be living with HIV are unaware of their infection and would not be included within the national cohort of persons accessing HIV care. However, the coverage of persons diagnosed and utilising HIV-related care indicates high rates of access to care and low rates of loss to follow-up [30]. Moreover, identifying additional HIV-positive individuals among IMD cases would only serve to strengthen the association between HIV and IMD, and increase the estimated relative risks further.

### Clinical implications

A protein-based, multi-component MenB vaccine has recently been licensed in Europe and will be introduced into the UK infant immunisation programme in 2015 [31]. This vaccine induces high concentrations of bactericidal antibodies against most MenB strains causing IMD in the UK and could potentially also protect against other meningococcal capsular groups [32]. HIV-infected infants born in the UK will, therefore, benefit from the vaccine programme, although there are currently very few infants infected through MTCT because of the high uptake of antenatal screening and very high effectiveness of antenatal and perinatal ART in preventing MTCT from mothers known to be HIV-positive [33]. Older children with HIV, including newly diagnosed entrants to the UK, would not be eligible for MenB vaccination because there is no catch-up programme planned for the UK. Reassuringly, though, invasive MenB disease is much less common in older children irrespective of HIV status.

In adolescents and young adults, the recent increase in invasive MenW disease in England caused by a hypervirulent clonal complex (cc11) clone has led to recommendations for a national adolescent MenACWY conjugate vaccination programme for 14–18 year-olds, which will be introduced imminently [34]. This programme should, therefore, also protect HIV-positive individuals of all ages against these four capsular groups through both direct and indirect (herd) protection. The potential high level of exposure to meningococcal for infected individuals born overseas, however, may be less amenable to prevention by a UK-based programme and may reflect a substantially higher risk in uninfected people with overseas origins.

The MenACWY conjugate vaccine, however, will not protect against MenB disease. Future studies on the immunogenicity of the recently licensed MenB vaccine in HIV-positive children and adolescents at different stages of immunosuppression and antiretroviral treatment could potentially support recommendations for MenB vaccination in these age groups. Our results also indicate that newly diagnosed adults with HIV may benefit from MenACWY conjugate vaccination [19, 35]. However, the wide range of CD4 counts, including a significant proportion with very low counts, and the variable duration and combinations of ART at the time of IMD diagnosis suggest that these parameters may not be useful criteria for recommending the most appropriate timing for meningococcal vaccination. Current UK guidelines suggest that vaccination should be given to persons with CD4 counts <200 cells/mm^3^ if indicated and safe, and repeated following immunoreconstitution if required [36].

There are three available MenACWY vaccines, each conjugated to a different carrier protein, and one (MenACWY-D; Menactra®, Sanofi Pasteur, Lyon, France) has been assessed in HIV-positive children and adolescents; the vaccine was immunogenic against the four capsular groups and a two-dose elicited higher bactericidal antibody concentrations and protected more individuals than a single dose [19, 37]. Vaccine responses were dependent on age, the presence of an AIDS-defining illness, CD4 count and HIV viral load [19, 35, 37], although none of these factors were consistently predictive of poorer antibody responses. Unlike the UK, a two-dose MenACWY primary MenACWY conjugate vaccine schedule, given at least 2 months apart, is recommended for HIV-positive adolescents (>11 years) in the US, with boosters every 5 years [38]; notably, the recently licensed MenB vaccines have been recommended for those considered at increased risk of MenB disease but not for HIV-positive individuals [39].

Another important observation was that HIV diagnosis was known at the time of IMD for nearly all cases. In only two patients was the diagnosis of HIV made at the time of IMD infection and, in one case, after recovering from IMD, highlighting the importance of considering an underlying diagnosis of HIV if clinically indicated, including but not limited to immigration from an HIV-endemic area, intravenous drug use or MSM, history of opportunistic infections or low lymphocyte counts when hospitalised for IMD.

### Availability of data and materials

All data for persons co-infected with HIV and invasive meningococcal disease are available within the Table 4. Further requests regarding individual participant data should be made to the clinical leads, Dr Shamez Ladhani for meningococcal (shamez.ladhani@phe.gov.uk) and Dr Valerie Delpech for HIV (valerie.delpech@phe.gov.uk).

## Conclusion

This study provides evidence that HIV-positive children and adults are at increased risk of IMD, up to five times higher than the general population. Furthermore, the risk among the capsular groups MenC, MenW, and MenY, preventable through the MenACWY quadrivalent conjugate vaccine, was 16 times higher in adults, especially 16-24 year-olds who had a much higher risk. These findings provide an evidence base for policy makers to include HIV as a risk factor for meningococcal vaccination, and clinicians to consider HIV as a possible underlying condition in children and adults who develop invasive meningococcal disease.

## Abbreviations

ART: antiretroviral therapy
BHIVA: British HIV Association
CI: confidence interval
GP: general practitioner
Hib: *Haemophilus influenzae* type b
HIV: human immunodeficiency virus
IMD: invasive meningococcal disease
IQR: interquartile range
MRU: Meningococcal Reference Unit
MSM: men who have sex with men
MTCT: mother-to-child transmission
NHS: National Health Service
PCR: polymerase chain reaction
PHE: Public Health England
PLHIV: people living with HIV/AIDS
